# Provision of ECPR during COVID-19: evidence, equity, and ethical dilemmas

**DOI:** 10.1186/s13054-020-03172-2

**Published:** 2020-07-27

**Authors:** Elliott Worku, Denzil Gill, Daniel Brodie, Roberto Lorusso, Alain Combes, Kiran Shekar

**Affiliations:** 1 Adult Intensive Care Services, The Prince Charles Hospital, Brisbane, Queensland Australia; 2 University of Queensland, Brisbane, Queensland Australia; 3 Center for Acute Respiratory Failure, New York-Presbyterian Hospital, New York, USA; 4 Columbia University College of Physicians and Surgeons/New York-Presbyterian Hospital, New York, USA; 5 Cardio-Thoracic Surgery Department, Heart and Vascular Centre, Maastricht University Medical Centre (MUMC), Cardiovascular Research Institute Maastricht (CARIM), Maastricht, The Netherlands; 6 Institute of Cardiometabolism and Nutrition, Sorbonne Universités, UPMC Univ Paris 06, 75651 Paris Cedex 13, France; 7 Medical Intensive Care Unit, Assistance Publique-Hôpitaux de Paris, Pitié-Salpêtrière Hospital, 75651 Paris Cedex 13, France; 8 Bond University, Gold Coast, Queensland Australia; 9 Critical Care Research Group and Centre of Research Excellence for Advanced Cardio-respiratory Therapies Improving OrgaN Support (ACTIONS), Brisbane, Australia

## Abstract

The use of extracorporeal cardiopulmonary resuscitation (ECPR) to restore circulation during cardiac arrest is a time-critical, resource-intensive intervention of unproven efficacy. The current COVID-19 pandemic has brought additional complexity and significant barriers to the ongoing provision and implementation of ECPR services. The logistics of patient selection, expedient cannulation, healthcare worker safety, and post-resuscitation care must be weighed against the ethical considerations of providing an intervention of contentious benefit at a time when critical care resources are being overwhelmed by pandemic demand.

## Introduction

Extracorporeal cardiopulmonary resuscitation (ECPR) describes the emergent use of extracorporeal membrane oxygenation (ECMO) to restore circulation in patients during cardiac arrest [1]. Optimal patient selection, timing of initiation, post-ECPR patient management, and logistical feasibility of providing an ECPR service remain ongoing challenges to securing good outcomes [2, 3]. Among patients suffering either an out-of-hospital or an in-hospital cardiac arrest (OHCA or IHCA), few meet established and generally agreed upon eligibility criteria, and even fewer can be successfully cannulated for ECMO within acceptable timeframes. This makes ECPR a low-volume, high-risk, and resource-intensive intervention, of contentious benefit. With mainly observational data to support the use of ECPR, much remains to be studied in the field.

The coronavirus disease 2019 (COVID-19) pandemic poses additional challenges to the safe and appropriate use of ECPR. Prioritising healthcare worker safety whilst facilitating expedient cannulation is fraught with complexity and presents a considerable barrier to the effective implementation of ECPR in this setting [4]. Appropriate candidate selection is a prerequisite for successful ECPR and is challenging under the best of circumstances [5]. Currently, identifying patients with the greatest potential to benefit from this resource-intense intervention is limited by the evolving understanding of the natural history of COVID-19 and the ability to prognosticate at an individual patient level. Finally, increased demand for critical care resources, the institution of crisis standards, and limitations on staffing and equipment are forcing the critical care community to confront the ethical boundaries between individual patient benefit, distributive justice, and resource allocation [6–8]. Rational decision-making must prevail in order to maximise both individual patient, and societal benefits.

## The rationale for ECPR

Interventions pertaining to the resuscitation of a patient in cardiac arrest are time critical. When cardiac arrest occurs, blood flow ceases and the resulting “no-flow” state rapidly produces irreversible neurological and multiorgan damage, if not promptly ameliorated. Conventional CPR (CCPR) produces a “low-flow” state that can temporarily sustain organ function. However, the longer the patient remains in cardiac arrest receiving CCPR, the less likely the patient is to achieve return of spontaneous circulation (ROSC), even when advanced life support measures are applied [9].

The application of ECMO to maintain organ perfusion is a well-established technique extrapolated from the use of cardiopulmonary bypass in cardiothoracic surgery, where an anesthetised patient is cannulated and mechanical circulatory support is initiated before the heart is arrested. In the case of ECPR, the patient is unconscious due to loss of cardiac output, and in this uncontrolled situation, the cannulation and establishment of mechanical circulatory support must occur rapidly. The American Heart Association (AHA), in their 2019 update, support consideration of ECPR for those failing CCPR, where it can be “expeditiously implemented, and supported by skilled providers” [10]. However, even with well-performed ECPR under non-pandemic conditions, the majority of patients will fail to survive with a good neurological outcome [11, 12].

**Fig. 1:**
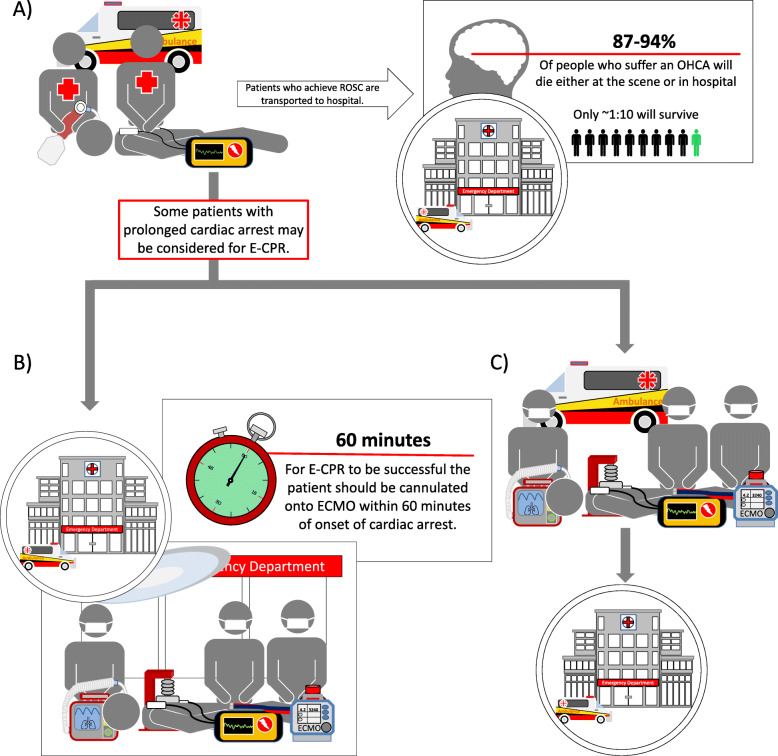
Current models for ECPR provision in OHCA. In all comers with OHCA, the vast majority will be pronounced dead at scene or on arrival to hospital. **a** In select patients with refractory cardiac arrest, ECPR may be advocated; this demands consideration of the predominant arrest rhythm (shockable preferable), the presence of bystander CPR, and the logistics of cannulation, ICU capacity, and availability of services such as PCI to determine and treat potential aetiologies. **b** Expedient cannulation and establishment of extracorporeal perfusion is a requisite of an effective ECPR; for OHCA, this may occur: (i) on-scene cannulation by mobile ECMO practitioners and (ii) rapid retrieval to ECPR hospital

There are currently two main models of ECPR provision [3] (Fig. 1). The first is *in-hospital cannulation*, whereby patients suffering an IHCA or OHCA who fail to achieve ROSC with standard CCPR and advanced cardiac life support (ACLS) may be cannulated for ECPR. The site of cannulation is often the emergency department (ED), or cardiac catheterisation laboratory for OHCA, or the intensive care unit (ICU), operating theatre, or cardiac catheterisation laboratory for patients with IHCA. The second is *pre-hospital cannulation* of patients suffering OHCA refractory to ACLS and who are attended at scene by a mobile ECPR cannulation team. Current data suggest that time to cannulation is a more important determinant of ECPR outcome than the site where cannulation occurs per se [13], and that stringent selection criteria applied rapidly at the scene may improve the yield of this intervention [14].

Identifying the group of patients who might benefit from ECPR is difficult. Having a reversible (predominantly cardiac) underlying aetiology for the arrest [3], the receipt of effective bystander CPR, the presenting arrest rhythm, and the time to initiation of ECPR [12] are important determinants of ECPR outcome [14]. Patients should also be free from precluding conditions, such as untreatable metastatic malignancy or life-limiting, end-stage, chronic disease [3].

The timing of transition from CCPR to the institution of ECPR is not universally agreed on. This conversion may be facilitated by the application of a mechanical CPR device to continue chest compressions whilst cannulation for ECPR is performed. Following restoration of organ perfusion with ECPR, a targeted intervention to address the underlying aetiology of arrest must be performed. Cardiac arrests of presumed cardiac origin have been disproportionately represented in ECPR studies; hence, high rates of angiography and subsequent percutaneous coronary intervention (PCI) are often seen [15]. This reflex resort to coronary angiography (intra-arrest PCI) may be challenged during the current pandemic, particularly in the absence of compelling ST elevation [16, 17].

## Performing ECPR during the COVID-19 pandemic

Severe acute respiratory syndrome coronavirus 2 (SARS-CoV-2) may lead to a multisystem illness, COVID-19, in many patients. The majority of infected individuals either are asymptomatic or suffer a mild respiratory tract infection. Approximately 15% of individuals with COVID-19 will develop a more severe illness, and 5–6% will develop critical illness characterised by severe respiratory failure with acute respiratory distress syndrome (ARDS) [18]. Additionally, SARS-CoV-2 can directly infect and impair other organ systems including the cardiac, gastrointestinal, renal, and central nervous systems, with the angiotensin-converting enzyme 2 (ACE2) receptor possibly implicated in viral tropism for these tissues. Other sequelae of COVID-19 disease may include a prothrombotic state, or immunodepression with superinfection, which in turn may potentiate acute pulmonary embolism, with ensuing acute cor pulmonale, or septic-like circulatory compromise. The number of critically unwell patients with COVID-19 who require ICU admission and organ support has overwhelmed health services in many countries globally, necessitating the rationing of critical care resources [19].

ECPR is complex, and efficient deployment relies on finely honed processes that may be significantly impacted by pandemic conditions. In acknowledging these circumstances, the current guidance on ECPR provided by ELSO [20] states the following:

- Centres with lesser experience or without established ECPR programmes are discouraged from initiating ECPR for OHCA during surge situations.
- Experienced centres may offer ECPR for IHCA for highly selected *non-COVID-19* patients depending on resource availability, whilst ECPR use in COVID-19-positive patients requires reflection on the risk-benefit ratio.
- Emergency conversion of venovenous ECMO to venoarterial ECMO in the setting of a cardiac arrest in a patient receiving venovenous ECMO or during cannulation is not recommended—due to the poor outcomes anticipated.

There are two scenarios that might be used to describe the aetiology of cardiac arrest during the COVID-19 pandemic. First, a cardiac arrest occurring in a patient who does not have COVID-19 would be presumed to be due to one of the currently understood aetiologies of OHCA and IHCA. These patients would be eligible for consideration of ECPR based on currently used criteria where resources are not constrained by the pandemic. The second is cardiac arrest in a patient who is known, or suspected, to have COVID-19. In this case, the aetiology of cardiac arrest may be related to the effects of the SARS-CoV-2 virus, as abovementioned, or the virus may simply be a bystander. In all circumstances, as community transmission of COVID-19 increases, it will become difficult to differentiate patients at presentation with respect to infectious status and the default will be a presumption of positivity. Irrespective of their infectious status and the aetiology of the cardiac arrest, the resource constraints imposed by the pandemic may limit usual processes of care. Delays in CCPR initiation due to the reluctance of members of the public and healthcare workers to initiate out-of-hospital resuscitation attempts, recognising those patients who might benefit from ECPR, requirements for donning personal protective equipment (PPE), impaired ambulance response times, and lack of critical care resources, may preclude the use of ECPR even in those who would otherwise be eligible.

Hypoxaemic respiratory failure leading to cardiac arrest appears to be common in COVID-19 patients. In a retrospective cohort study of 136 patients suffering IHCA in Wuhan, China, 87.5% of arrests were due to a respiratory aetiology. The vast majority occurred outside of an ICU setting, and shockable rhythms were observed in only 5.9% of this cohort. Survival outcomes were dismal in this cohort with only one patient surviving to 30 days with a favourable neurologic outcome (Cerebral Performance Category (CPC) [1, 2, 21]). It is also reported that the interplay between patient comorbidities, in particular cardiovascular risk factors, and the aetiologic virus may give rise to a range of cardiovascular pathological insults [22]. Acute myocardial infarction, myocarditis, and coronary spasm fuelled by hyperinflammation, multiorgan dysfunction, thrombotic phenomena, and severe hypoxaemia resulting in cardiac arrest have been described [23]. Cardiac arrhythmias are also frequently reported and may reflect direct effects of the disease or cardiotoxicity from agents [24] repurposed to treat COVID-19.

In a true surge crisis, critical care demand outstrips capacity, and it becomes untenable to provide ECPR and post-resuscitative support. Establishing even basic CCPR may be hindered by delayed response times, time to allow PPE donning, and system pressures diverting the resuscitation team. Exceptions would include patients who arrest during coronary angiography, for instance, in which case rapid cannulation is possible, the aetiology may be more amenable to reversal, and there is the added benefit of advanced imaging available to confirm cannula placement should ECPR be initiated. Continuing access to ECPR during the COIVD-19 pandemic requires adherence to surge-specific protocols and prompt involvement of senior decision-makers at the time of an arrest in order to promote acceptable outcomes. Local tools could be developed to aid with rapid assessment of ECPR candidacy and feasibility [3].

## Modifications to establishing ECPR

**Fig. 2:**
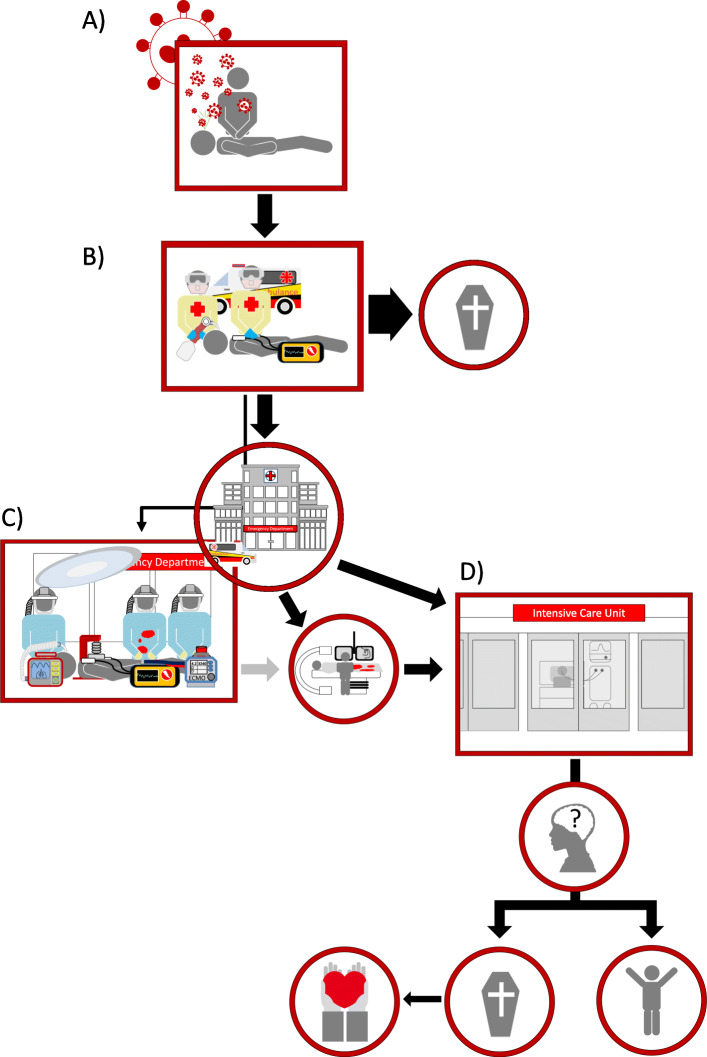
Possible management of the confirmed or suspected COVID-19-positive patient with OOHCA. **a** Bystander CCPR, with risk of aerosolisation and viral transmission: in many cases, this may not be performed on patients with known infectious status. **b** Ambulance service provides defibrillation and early airway securement to minimise aerosol generation. Time to don PPE and elevated system demands may delay attendance. *In sustained non-shockable cardiac arrest, it may be appropriate to curtail resuscitation and avoid hospital transfer.***c** E-CPR if appropriate, in an isolated negative pressure environment with mechanical compressions. ECMO team should be in high-level PPE including PAPR. *In non-ECPR centres, the patient may proceed to coronary angiography if appropriate intra-arrest or more typically post-ROSC. Inter-hospital transfer for ECPR or PCI would not be routine.***d** ICU admission is contingent upon patient prognosis and system capacity*. It may be reasonable to admit only if ROSC has been achieved*. Good neurological survival remains the desired outcome. Patients may receive TTM/hypothermia and ongoing mechanical circulatory support for an agreed duration. Outcomes include recovery, WLST, or brain death*. Organ donation may only be considered in patients confirmed to be COVID-19 negative*. CCPR, compression only CPR; PPE, personal protective equipment; TTM, targeted temperature management; WLST, withdrawal of life-sustaining therapy

As with CCPR, ad hoc decision-making regarding ECPR should be discouraged. Goals of care and resuscitation status should be addressed transparently with patients and surrogate decision-makers, so that any limitations dictated by patient or system factors are explicit. The nature of ECPR often precludes such discussions from happening in real-time, only adding to the burden of responsibility on clinicians. The ECPR team response in COVID-19 (Fig. 2) is complicated by the need for PPE arising from the heightened risk of healthcare worker infection and contamination of clinical areas and equipment by aerosolised fomites [25] and blood [26].

Provision of ECPR should ideally be an interdisciplinary decision and is best prepared for via high-fidelity simulation and streamlined cannulation teams [4, 20]. For example, situations such as ECPR in the COVID-19 patient who is in the prone position would need to be anticipated and rehearsed, if such a scenario is to be considered. There also needs to be an appreciation for wider system demands. For example, there are critical blood product shortages (due in part to a shrinking pool of healthy donors), and so conservation strategies such as percutaneous over surgical cannulation are important [27]. Patient transfer after ECPR necessitates predesignated egress routes from the place of cannulation to other destinations, to mitigate the risks of cross-contamination of “clean” areas. Ideally, in already experienced ECMO centres, patients treated for COVID-19 disease and related complications should have a clearly declared escalation status, including candidacy for ECMO support and ECPR if the need were to arise. Such decisions must take into account the local protocols, the equipment availability, and the readiness of the system to accommodate sudden and dramatically increased demands.

## Ethical considerations

A fundamental principle underpinning all pandemic responses is the maximisation of benefit from scarce resources [6]. Resource scarcity and resource *saturation* are fluid judgements, relying on continual cycles of appraisal, integrating real-time data, and epidemiological projections [8]. Maximising benefit refers not only to enhancing survival in individuals, but also to extending this opportunity to as many patients as possible or most appropriate candidates to benefit from. ECPR exacts a heavy toll on equipment (membrane oxygenator and ECMO circuit, blood products, ultrasound devices, and PPE among them) and staff (intensified nursing and other key supports, ECMO specialists, senior intensivist oversight, and others). Personnel siphoned to support ECPR might be better deployed caring for several other less critical patients with a better chance of helping a greater number; similarly, the ECMO circuit may provide respiratory support for a patient with single organ dysfunction.

Beyond the immediate intervention, there are ongoing costs to convalescence. Although survivors of ECPR may demonstrate favourable neurologic outcome, the COVID-19 cohort cannot be assumed to be typical. The potential for generating dependent survivors is a burden ECPR may impose, and the benefits of ECPR may be overestimated in the COVID-19 cohort. There may be survivors with ECPR during the COIVD-19 pandemic, but at what individual and health opportunity cost? If resources are committed to ECPR early during a surge, ongoing availability of ICU resources may be further limited to other patients, infected or not [28], who have a greater probability of benefit. Whilst it is important to support the individuals’ right to treatment, equity during a pandemic dictates a process of triage and prioritisation, ensuring that healthcare allocation is contingent on anticipated utility and maximum benefit. Many scenarios may arise without a clear right answer. Best judgement necessarily depends on dispassionate, open communication, triage, and frequent re-evaluation of the healthcare landscape.

The right to withhold life-sustaining treatment varies globally [29]. In Denmark, CCPR initiation by paramedics is a mandatory practice as the absence of a circulation defines a patient *with cardiac arrest*, not death [30]. In some jurisdictions, it is only when “inconsistent with good medical practice” that it is permissible to withdraw therapy without consent [31]. With respect to life-sustaining treatment, the concept of futility is ill-defined, and often there is poor agreement between the physician and patient or surrogate decision-maker. Furthermore, public perception of CCPR is skewed through media portrayals. The most appropriate argument for withholding or withdrawing CCPR and ECPR in COVID-19 patients must be non-maleficence to the patient and others. It is accepted that extended resuscitation can be curtailed during crises, and since ECPR is not yet the standard of resuscitative care, whether access to ECPR may be refused is not nearly as contentious as reluctance to provide CCPR. A number of health systems are declining resuscitation of COVID-19-positive patients as a rule [32], discerning risks to the system and healthcare workers from potential aerosolisation to exceed individual benefit of attempted resuscitation. Although resuscitation is sometimes performed to alleviate family suffering, by providing assurances that “everything was done”, this practice should be questioned during a crisis. Emotions should be tempered, and objectivity should dictate actions [30].

### Withdrawal of ECMO support

Some centres routinely mandate ethicist consultations in the withdrawal of ECMO support [33]. The urgency of ECPR necessitates expediency, so limited opportunities exist to fully explore outcome scenarios with surrogate decision-makers at the time of cannulation; the decision to offer and subsequently limit ECPR rests heavily on the clinicians’ shoulders. ECPR initiation is an organisational decision, requiring support from multiple specialties. This impacts on attendance to other clinical priorities, risks to other hospitalised patients, and to the hospital infrastructure; therefore, cannulation should be by consensus; equally, withdrawal should incorporate multiple stakeholders. Fairness and equity dictate that objective criteria motivate both treatment escalation and withdrawal of life-sustaining therapies. Where ECPR has been offered, evolving multiorgan dysfunction or signs of poor neurological recovery must prompt the treating team to approach the subject of withdrawal [2], in systems where this is considered acceptable, not only to prevent burdensome treatment to the patient, but also to make available resources for those who may yet benefit. These hard truths would ideally be explicit before cannulation commences and will require empathy and transparency with families [33]. Staff morale is an important but secondary consideration, and both moral and psychological injuries [34] to the workforce are significant risks when embarking upon interventions with limited potential for therapeutic benefit.

## Conclusion

Observational data suggests that ECPR provides an improved opportunity for favourable neurological survival in highly selected patients experiencing cardiac arrest compared with CCPR. Whilst there is a need for prospective study and high-quality randomised trials in this area [35], the current COVID-19 pandemic presents practical and ethical challenges to the ongoing provision and implementation ECPR programmes. At a time when critical care faces heavy constraints, it is important to work within ethical and legal frameworks to espouse equity and consistency in healthcare allocation, remembering not to inadvertently disadvantage non-COVID-19 patients.

## Data Availability

Not applicable.
